# RETRACTION: “Effect of Interleukin‑12 Gene Expression on Insulin Resistance in Patients With Acne Vulgaris”

**DOI:** 10.1111/srt.70355

**Published:** 2026-04-24

**RETRACTION**: A. AbdElneam, M. S. Al‐Dhubaibi, S. S. Bahaj, G. F. Mohammed, A. K. Alantry, and L. M. Atef, “Effect of Interleukin‑12 Gene Expression on Insulin Resistance in Patients With Acne Vulgaris,” *Skin Research and Technology* 29, no. 11 (2023): e13503, https://doi.org/10.1111/srt.13503.

The above article, published online on 24 October 2023 in Wiley Online Library (wileyonlinelibrary.com), has been retracted by agreement between *Skin Research and Technology*; and John Wiley & Sons Ltd. The article was accepted for publication following a compromised peer review process. Furthermore, the study's rationale was found to be unsupported by the cited literature, as the references cited in support of this rationale did not substantiate the statements for which they were referenced. Finally, the reported values used to assess insulin resistance were found to be physiologically implausible, indicating methodological or analytical errors that render the findings unreliable. Accordingly, the article has been retracted. The authors disagreed with this decision.

